# Declines in SARS-CoV-2 Transmission, Hospitalizations, and Mortality After Implementation of Mitigation Measures— Delaware, March–June 2020

**DOI:** 10.15585/mmwr.mm6945e1

**Published:** 2020-11-13

**Authors:** Florence A. Kanu, Erica E. Smith, Tabatha Offutt-Powell, Rick Hong, Delaware Case Investigation and Contact Tracing Teams, Thu-Ha Dinh, Eric Pevzner

**Affiliations:** ^1^Epidemic Intelligence Service, CDC; ^2^CDC COVID-19 Response Team; ^3^COVID-19 Outbreak Response Team, Division of Public Health, Delaware Department of Health and Social Services.

*On November 6, 2020, this report was posted as an *MMWR* Early Release on the MMWR website (*https://www.cdc.gov/mmwr*).*

Mitigation measures, including stay-at-home orders and public mask wearing, together with routine public health interventions such as case investigation with contact tracing and immediate self-quarantine after exposure, are recommended to prevent and control the transmission of SARS-CoV-2, the virus that causes coronavirus disease 2019 (COVID-19) (*1*–*3*). On March 11, the first COVID-19 case in Delaware was reported to the Delaware Division of Public Health (DPH). The state responded to ongoing community transmission with investigation of all identified cases (commencing March 11), issuance of statewide stay-at-home orders (March 24–June 1), a statewide public mask mandate (from April 28), and contact tracing (starting May 12). The relationship among implementation of mitigation strategies, case investigations, and contact tracing and COVID-19 incidence and associated hospitalization and mortality was examined during March–June 2020. Incidence declined by 82%, hospitalization by 88%, and mortality by 100% from late April to June 2020, as the mask mandate and contact tracing were added to case investigations and the stay-at-home order. Among 9,762 laboratory-confirmed COVID-19 cases reported during March 11–June 25, 2020, two thirds (6,527; 67%) of patients were interviewed, and 5,823 (60%) reported completing isolation. Among 2,834 contacts reported, 882 (31%) were interviewed and among these contacts, 721 (82%) reported completing quarantine. Implementation of mitigation measures, including mandated mask use coupled with public health interventions, was followed by reductions in COVID-19 incidence and associated hospitalizations and mortality. The combination of state-mandated community mitigation efforts and routine public health interventions can reduce the occurrence of new COVID-19 cases, hospitalizations, and deaths.

Using laboratory and case investigation data, changes in COVID-19 incidence and associated hospitalization and mortality in Delaware during March 11–June 25 were assessed. Laboratory data from the Delaware Electronic Reporting and Surveillance System (DERSS) and case investigation data from Delaware DPH were obtained. DERSS data included case classification (e.g., laboratory-confirmed or probable*); case investigation data included hospitalization status and outcome, including death. Incidence was defined as the number of newly confirmed COVID-19 patients per 10,000 Delaware residents per week (*4*). Hospitalization and mortality rates were calculated similarly, as the number of patients with confirmed COVID-19 who were hospitalized or died per 10,000 persons per week. Percent change was calculated to describe the magnitude of rate change. Delaware mitigation and public health interventions included 1) case investigations (starting March 11), which involved interviewing patients with SARS-CoV-2 infection, asking each to immediately self-isolate while collecting information on demographic characteristics, potential exposure source, symptoms,^†^ and close contacts^§^; 2) statewide mandated stay-at-home order^¶^ (March 24–June 1); 3) statewide mandated mask use in public** (instituted April 28); and 4) contact tracing (starting May 12), wherein close contacts were interviewed and asked to self-quarantine and report symptoms for 14 days following an exposure. Changes in COVID-19 incidence and associated hospitalizations and mortality from March through June were assessed.

After the initial case report and stay-at-home order, COVID-19 incidence, hospitalization, and mortality rates increased, peaking during the week of April 13 at 15.0, 2.0, and 0.8 per 10,000 persons, respectively (Figure). After the peak, incidence declined by 18%, hospitalizations by 20%, and deaths by 13%, before increasing slightly during the week of April 20. Rates declined again the same week the mask mandate went into effect (April 28) and continued to decline by 82% (incidence), 88% (hospitalization) and 100% (mortality) from late April through June, as contact tracing was added to case investigations, the stay-at-home order, and the mask mandate.

**FIGURE:**
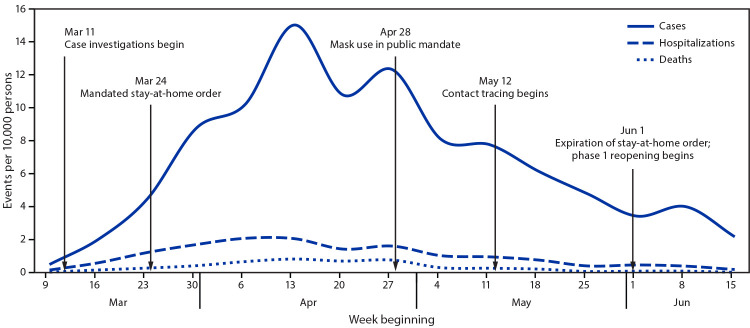
Confirmed COVID-19 cases, associated hospitalizations, and deaths reported to Delaware Division of Public Health, by week, and COVID-19 mitigation efforts — Delaware Department of Health and Social Services, March 9–June 15, 2020 **Abbreviation:** COVID-19 = coronavirus disease 2019.

During March 11–June 25, a total of 9,762 newly confirmed COVID-19 cases were identified in DERSS; among these cases, 6,527 (67%) patients were interviewed and asked to self-isolate, among whom 5,823 (89%) had been released from isolation^††^ at the time of data collection. Median patient age was 41 years (interquartile range [IQR] = 28–54 years), and 55% were female (Table). The median interval from receiving a positive test result to interview was 8 days (IQR = 6–12 days) and from DPH’s receipt of case report to interview was 5 days (IQR = 2–8 days). Patients who were not interviewed were those who did not respond to call attempts (1,134, 12%), were in a hospital/long-term care facility at time of contact (788, 8%), did not have an available phone number (673, 7%), had died (433, 4%), or were not interviewed for other reasons^¶¶^ (207, 2%). Among interviewed patients, 5,742 (88%) reported having any COVID-19–related symptoms before the interview date, and 55% reported close contact with someone with diagnosed COVID-19.

**TABLE:** Characteristics of persons with confirmed COVID-19 (patients) and contacts interviewed during case and contact investigations — Delaware, March–June 2020

Characteristic	No. (%)	
	Interviewed patients (n = 6,527)	Interviewed contacts (n = 882)
**Age, yrs, median (IQR)**	41 (28–54)	25 (14–74)
**Time from positive COVID-19 test to interview, days, median (IQR)**	8 (6–12)	N/A
**Time from report to interview, days, median (IQR)**	5 (2–8)	2 (1–4)
**Sex**		
Male	2,911 (44.6)	449 (50.9)
Female	3,614 (55.4)	433 (49.1)
Missing	2 (0.03)	0 (—)
**Race/Ethnicity**		
White, non-Hispanic	1,769 (27.1)	202 (22.9)
Black or African American, non-Hispanic	1,949 (29.9)	155 (17.6)
Hispanic/Latino	2,237 (34.3)	350 (39.7)
Asian, non-Hispanic	128 (2.0)	6 (0.7)
Other/Multiple races, non-Hispanic	327 (5.0)	20 (2.3)
Unknown/Missing	117 (1.8)	149 (16.9)
Any symptoms*		
No	753 (11.5)	721 (81.8)
Yes	5,742 (88.0)	161 (18.3)
Unknown/Missing	32 (0.5)	0 (—)
**Close contact with confirmed COVID-19 case**		
No	2,222 (34.0)	N/A
Yes	3,574 (54.8)	882 (100)
Unknown/Missing	731 (11.2)	0 (—)
**Household exposure^†^ to known COVID-19 case**		
No	1,235 (19.0)	100 (11.3)
Yes	2,039 (31.3)	771 (87.4)
Unknown/Missing	3,253 (49.8)	11 (1.3)
**Hospitalized**		
No	5,606 (85.9)	N/A
Yes	742 (11.4)	N/A
Unknown/Missing	179 (2.7)	N/A
**Died from COVID-19**		
No	6,477 (99.2)	N/A
Yes	14 (0.2)	N/A
Unknown/Missing	36 (0.6)	N/A

**Sources:** Delaware Case Investigation and Contract Tracing Systems, Delaware Department of Health and Social Services, Division of Public Health.

**Abbreviations:** COVID-19 = coronavirus disease 2019; IQR = interquartile range; N/A = not applicable.

* Patients were asked if they had symptoms from 14 days before test date to date of interview; contacts were asked during monitoring period if they had symptoms.

^†^ Household exposure defined as possible exposure to COVID-19 from a household member with confirmed COVID-19.

Among 6,527 interviewed patients with laboratory-confirmed COVID-19, 5,390 (83%) either refused to name contacts or could not recall contacts. The mean number of contacts reported per patient who reported one or more contacts was 2.5 (IQR = 1–3). Among the 2,834 contacts reported, complete contact information was obtained for 1,869 (66%), and 882 (47%) of those were interviewed and asked to self-quarantine. The median interval from patient interview to contact interview was 2 days (IQR = 1–4 days). The median age of interviewed contacts was 25 years (IQR = 14–47 years), 433 (49%) were female, 721 (82%) did not develop symptoms during quarantine, and 771 (87%) lived in the same household as someone with confirmed COVID-19. Overall, 161 (18%) of the 882 contacts who were reached experienced symptoms during quarantine and were urged to be tested for SARS-CoV-2. A manual search of DERSS data determined that among 161 symptomatic contacts, 20 (12%) were tested, four of whom (3%) received a COVID-19 diagnosis. Reasons for not interviewing contacts included that the contact did not respond to call attempts (265, 14%), had no available phone number (208, 11%), refused (88, 5%), was a non-Delaware resident (20, 1%), or other reasons (406, 22%).

## Discussion

A stay-at-home order and case investigations instituted weeks before the peak in COVID-19 cases (week of April 13) in Delaware likely contributed to the subsequent decline observed in COVID-19 incidence and associated hospitalization and deaths. As expected, the impact on incidence was not immediate but occurred weeks after measures were implemented, as new cases represented exposure that occurred during previous weeks. Additional steep declines in reports of new cases occurred after a public mask use mandate was issued in late April. Masks are critical for reducing SARS-CoV-2 transmission from persons with symptomatic or asymptomatic infection (*5*). Wearing masks can prevent respiratory droplets containing SARS-CoV-2 from traveling into the air and being transmitted to other persons and thus can reduce exposures and infections (*6*,*7*).

Early detection, self-isolation, and investigation of COVID-19 cases and self-quarantine of close contacts can be effective in preventing community transmission, if contacts are identified and reached soon after exposure (*8*). Because of limited resources and the growing number of cases, contact tracing in Delaware officially began in May when the Delaware National Guard was activated to assist Delaware DPH. In Delaware, contacts were monitored until symptom onset or quarantine completion. Testing was recommended for contacts reporting symptoms; however, no active follow-up was performed because of constrained resources. Case investigation was completed among contacts who received positive test results; therefore, having active follow-up and referral systems for testing contacts could expand disease prevention and containment opportunities.

Several barriers to case investigation and contact tracing were identified. First, low numbers of contacts were identified: 83% of interviewed patients either refused to disclose contacts or could not recall contacts. Second, cases were contacted a median of 8 days after receiving their positive test result and 5 days after report of this result to DPH. Earlier initiation of case investigation might increase recall and early identification of close contacts and thus prevent further disease transmission. Lastly, 22% of contacts could not be reached for reasons designated as “other,” an interaction outcome in case investigations and contact tracing reserved for circumstances interviewers could not address without additional information. Daily and weekly data monitoring to provide additional information for those with “other” as an interaction outcome could increase the number of persons reached. These barriers to contact tracing might have limited effectiveness and were missed opportunities to recommend other mitigation strategies (e.g., testing, quarantine, or isolation).

The findings in this report are subject to at least three limitations. First, data on adherence to the state-mandated stay-at-home order and use of masks in public were not available. Second, adherence to self-quarantine was self-reported. Finally, because of the observational design of the study, the decline in COVID-19 incidence, hospitalization, and mortality could not be attributed to the relative contribution of each mitigation measure.

A combination of mitigation measures including stay-at-home orders, mandated mask use in public, and case investigations with contact tracing, can reduce COVID-19 incidence and associated deaths (*9*). No single mitigation strategy is likely to be effective alone. These strategies are effective in limiting potential exposure to SARS-CoV-2 and reducing community transmission when implemented as part of a multicomponent strategy (*10*). In Delaware, state-mandated community mitigation efforts, such as stay-at-home orders, coupled with mask use, likely contributed to the decline in new COVID-19 cases. SARS-CoV-2 community transmission, hospitalization, and mortality can be reduced with statewide mitigation strategies implemented in tandem with the routine public health interventions of case investigation with contact tracing, and immediate self-isolation of cases and self-quarantine of contacts.

**Summary**

**What is already known about this topic?**

COVID-19 mitigation measures (e.g., stay-at-home orders and public mask mandate) and fundamental public health interventions (e.g., case investigations and contact tracing with prompt isolation or quarantine) are primary approaches to preventing and controlling SARS-CoV-2 community transmission.

**What is added by this report?**

State-mandated stay-at-home orders and public mask mandates coupled with case investigations with contact tracing contributed to an 82% reduction in COVID-19 incidence, 88% reduction in hospitalizations, and 100% reduction in mortality in Delaware during late April–June.

**What are the implications for public health practice?**

The combination of state-mandated community mitigation efforts and routine public health interventions can reduce the occurrence of new COVID-19 cases, hospitalizations, and deaths.

## Footnotes

* A probable case was defined as an illness meeting clinical criteria and having an epidemiologic link with no confirmatory laboratory testing performed for COVID-19, or an illness with presumptive laboratory evidence (detection of antigen or antibody) and either meeting clinical criteria or having an epidemiologic link, or an illness in a person meeting vital records criteria with no confirmatory laboratory testing for COVID-19 performed. https://www.cdc.gov/coronavirus/2019-ncov/covid-data/faq-surveillance.html.

† The following signs and symptoms were monitored: cough, fever, fatigue, body aches, loss of taste/smell, shortness of breath, chills, and sore throat.

§ Case investigation included inquiries about close contacts in May 2020. At time of data collection, CDC guidance on contact definition was “persons less than 6 feet from a confirmed case for at least 10 minutes.” CDC guidance now defines contacts as any person within 6 feet of an infected person for a cumulative total of 15 minutes or more over a 24-hour period starting from 2 days before illness onset (or, for asymptomatic patients, 2 days prior to test specimen collection). https://www.cdc.gov/coronavirus/2019-ncov/php/contact-tracing/contact-tracing-plan/appendix.html#contact.

¶ https://governor.delaware.gov/health-soe/fifth-state-of-emergency/.

** https://governor.delaware.gov/health-soe/thirteenth-state-of-emergency/.

†† Release from isolation was dependent on CDC guidance during this study period: initially, patients were asked to isolate for 14 days from symptom onset or test date and be symptom-free for 7 days. Later, patients were asked to isolate for 10 days from symptom onset or test date and be symptom-free for 3 days. Patients with asymptomatic cases were asked to isolate for 10 days after test date.

¶¶ Other status was reserved for unique circumstances that could not be addressed by case investigators or contact tracers without additional information.
